# Supplementary material for: The carbohydrate-binding module of TrCel7A aids in navigating hemicellulose barriers in plant cell walls
Source: J Biol Chem. 2026 Jun 1;302(7):113218. doi: 10.1016/j.jbc.2026.113218 (PMC13315674; doi:10.1016/j.jbc.2026.113218)
Supplement: Supporting Figure and Tables [file mmc1.pdf]

Supporting information for Zexer et al., The Carbohydrate Binding Module of TrCel7A Aids in Navigating Hemicellulose Barriers in Plant Cell Walls

		BC	PASC	MWC	hCNF
Cel7A	k_{fast}	0.099 ± 0.0.052	0.069 ± 0.023	0.045 ± 0.03	0.041 ± 0.03
	k_{slow}	0.012 ± 0.04	0.017 ± 0.035	0.006 ± 0.003	0.009 ± 0.003
	$\boldsymbol{k_{avg}}$	**0.044 ± 0.018**	**0.04 ± 0.007**	**0.033 ± 0.013**	**0.034 ± 0.019**
Cel7A$^{\Delta CBM}$	k_{fast}	0.025 ± 0.009	0.028 ± 0.026	0.022 ± 0.006	0.013 ± 0.005
	k_{slow}	0.009 ± 0.005	0.005 ± 0.03	0.004 ± 0.0.002	0.005 ± 0.005
	$\boldsymbol{k_{avg}}$	**0.013 ± 0.002**	**0.008 ± 0.004**	**0.007 ± 0.001**	**0.004 ± 0.003**

Table S1: Comparison of weighted average rate constants (k_{avg}), fast binding rate constants (k_{fast}), and slow binding rate constants (k_{slow}) for Cel7A and Cel7A$^{\Delta CBM}$ on BC, PASC, MWC, and hCNF. Values (in s^{-1}) are presented as mean ± standard deviation from at least three independent experiments.

	hCNF	hCNF-KOH
Glucose	79 %	100 %
Xylose	15 %	-
Fucose<br>Arabinose<br>Rhamnose<br>Galactose<br>Mannose	6 %	-

Table S2: Relative sugar content as found by monosaccharide analysis of holocellulose (hCNF) and alkali-treated holocellulose (HCNF-KOH). For the hCNF, Fucose, Arabinose, Rhamnose, Galactose, and Mannose together constitute 6% of the total sugar. Values are represented as percentage of total identified sugars. (-), not detected.

substrate	enzyme / domain	k_{obs}	k_{off}	k_{on}
BC	Cel7A	0.044 ± 0.018	0.009 ± 0.001	0.035 ± 0.018
	Cel7A$^{\Delta CBM}$	0.013 ± 0.002	0.009 ± 0.002	0.004 ± 0.003
PASC	Cel7A	0.04 ± 0.007	0.004 ± 0.001	0.036 ± 0.007
	Cel7A$^{\Delta CBM}$	0.008± 0.004	0.005 ± 0.002	0.003 ± 0.004
MWC	Cel7A	0.033 ± 0.013	0.007 ± 0.001	0.026 ± 0.013
	Cel7A$^{\Delta CBM}$	0.007 ± 0.001	0.006 ± 0.002	0.001 ± 0.002
hCNF	Cel7A	0.034 ± 0.019	0.008 ± 0.002	0.026 ± 0.019
	Cel7A$^{\Delta CBM}$	0.004 ± 0.003	-	-
hCNF-KOH	Cel7A	0.013 ± 0.003	0.004 ± 0.001	0.009 ± 0.003
	Cel7A$^{\Delta CBM}$	0.007 ± 0.003	0.003 ± 0.001	0.004 ± 0.003

Table S3: Measured binding rate constants (k_{obs}), measured off-rate constants (k_{off}), and calculated on-rate constants (k_{on}; all in s^{-1}) for Cel7A and Cel7A$^{\Delta CBM}$ on different substrates. On-rate is calculated from $k_{obs} = k_{on}$*[Cel7A] + k_{off} where [Cel7A] is 1 nM in all experiments. Off-rate is corrected for photobleaching. At least three separate experiments were used to determine each k_{obs} and k_{off}. Due to low particle number, k_{off} could not be measured for hCNF.

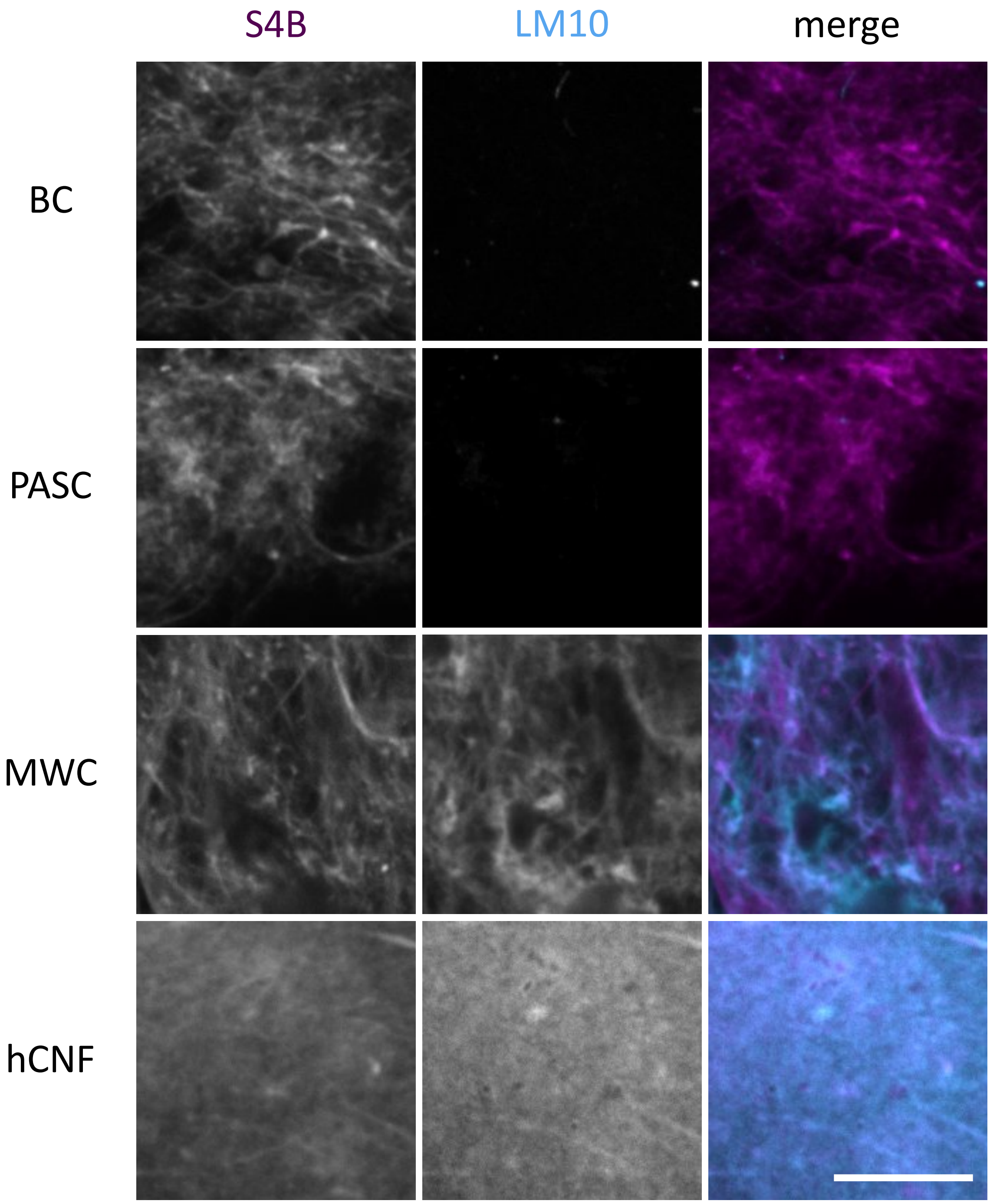

Figure S1. Detection of xylan in substrates used in this study. Cellulose staining using S4B (left column) and xylan immunofluorescence using LM10 antibodies and Alexa 488-labeled secondary antibody (middle column). Merged images show the S4B signal in magenta and the Alexa 488 signal in cyan (right column). Bacterial Cellulose (BC), Phosphoric Acid Swollen Cellulose (PASC), Milkweed Cellulose (MWC) and Holocellulose Nanofibrils (hCNF). Scale bar common to all panels is 20 μm.
